# Supplementary material for: Temporary Right-Ventricular Assist Devices: A Systematic Review
Source: J Clin Med. 2022 Jan 26;11(3):613. doi: 10.3390/jcm11030613 (PMC8837135; doi:10.3390/jcm11030613)
Supplement: Supplementary file 1 [file jcm-11-00613-s001.zip › jcm-1532310-supplementary.pdf]

Supplementary Table S1. Metadata of the 31 studies included in the systematic review

Author	Year	Country	Journal	Type	Duration
Aissaoui et al.[1]	2014	Germany	American Society for Artificial Internal Organs Journal	RS	January 2000 to May 2011
Anderson et al.[2]	2018	US	The Journal of Heart and Lung Transplantation	PM	NR
Badu et al.[3]	2020	US	American Society for Artificial Internal Organs Journal	RS	April 2017 to February 2019
Bhama et al.[4]	2018	US	The Journal of Thoracic and Cardiovascular Surgery	RS	Between 2005 and 2013
Cheung et al.[5]	2014	Canada	The Journal of Heart and Lung Transplantation	RM	Between 2005 and 2011
Coromilas et al.[6]	2019	US	Journal of Cardiac Failure	RS	March 2007 to April 2018
Deschka et al.[7]	2016	Germany	Journal of Cardiothoracic and Vascular Anesthesia	RM	February 2011 to February 2015
Jaidka et al.[8]	2019	Canada	Canadian Journal of Cardiology Open	RS	January 2015 to December 2017
Kapur et al.[9]	2013	US	JACC: Heart Failure	RM	Between 2007 and 2011
Khani-Hanjani et al.[10]	2013	US	American Society for Artificial Internal Organs Journal	RS	October 2009 to September 2011
Khorsandi et al.[11]	2019	US	The Annals of Thoracic Surgery	RS	October 2007 to October 2017
Kiernan et al.[12]	2017	INTERMACS	Circulation. Heart Failure	RM	June 2006 to March 2014
Kremer et al.[13]	2020	Germany	European Society of Cardiology Heart Failure Journal	RS	July 2016 to November 2019
Lazar et al.[14]	2013	US	The Annals of Thoracic Surgery	RS	Between 2006 and 2011
Leidenfrost et al.[15]	2016	US	European Journal of Cardio-Thoracic Surgery	RS	March 2009 to June 2013
Loforte et al.[16]	2013	Italy-Germany	Journal of Artificial Organs	RM	Between 2007 and 2011
Loforte et al.[17]	2011	Italy	American Society for Artificial Internal Organs Journal	RS	February 2004 and May 2010
Oliveros et al.[18]	2021	US	Journal of Invasive Cardiology	RS	August 2015 to February 2018
Patil et al.[19]	2015	UK	The Journal of Thoracic and Cardiovascular Surgery	RS	July 2003 to June 2013

Author	Year	Country	Journal	Type	Duration
Qureshi et al.[20]	2020	US	Catheterization and Cardiovascular Interventions	RM	June 2016 to April 2018
Ravichandran et al.[21]	2018	US	American Society for Artificial Internal Organs Journal	RS	October 2014 to March 2016
Saeed et al.[22]	2015	Germany	The Journal of Thoracic and Cardiovascular Surgery	RS	January 2010 to Feb 2014
Saito et al.[23]	2012	Japan	The Journal of Heart and Lung Transplantation	RS	January 2001 to December 2010
Salana et al.[24]	2020	US	Interactive CardioVascular and Thoracic Surgery	RS	January 2016 to March 2019
Schaefer et al.[25]	2017	Germany	American Society for Artificial Internal Organs Journal	RS	January 2012 to April 2016
Schmack et al.[26]	2019	Germany	Journal of Thoracic Disease	RS	October 2015 to September 2017
Schopka et al.[27]	2012	Germany	Journal of Artificial Organs	RS	Between 2006 and 2011
Shekiladze et al.[28]	2020	US	Catheterization and Cardiovascular Interventions	RS	January 2016 to December 2018
Takeda et al.[29]	2014	US	The Journal of Heart and Lung Transplantation	RS	January 2000 to December 2012
Vierecke et al.[30]	2019	EUROMACS	European Journal of Cardio-Thoracic Surgery	RM	January 2011 to 21 October 2017
Yoshioka et al.[31]	2017	US	Interactive CardioVascular and Thoracic Surgery	RS	January 2009 to December 2014.

References

1. Aissaoui, N.; Morshuis, M.; Paluszkiewicz, L.; Lauenroth, V.; Börgermann, J.; Gummert, J. Comparison of biventricular and left ventricular assist devices for the management of severe right ventricular dysfunction in patients with end-stage heart failure. *ASAIO J* **2014**, *60*, 400-406.
2. Anderson, M.; Morris, D.L.; Tang, D.; Batsides, G.; Kirtane, A.; Hanson, I.; Meraj, P.; Kapur, N.K.; O'Neill, W. Outcomes of patients with right ventricular failure requiring short-term hemodynamic support with the Impella RP device. *J Heart Lung Transplant* **2018**, *37*, 1448-1458.
3. Badu, B.; Cain, M.T.; Durham, L.A.; Joyce, L.D.; Sundararajan, S.; Gaglianello, N.; Ishizawar, D.; Saltzberg, M.; Mohammed, A.; Joyce, D.L. A Dual-Lumen Percutaneous Cannula for Managing Refractory Right Ventricular Failure. *ASAIO J* **2020**, *66*, 915-921.
4. Bhama, J.K.; Bansal, U.; Winger, D.G.; Teuteberg, J.J.; Bermudez, C.; Kormos, R.L.; Bansal, A. Clinical experience with temporary right ventricular mechanical circulatory support. *J Thorac Cardiovasc Surg* **2018**, *156*, 1885-1891.
5. Cheung, A.W.; White, C.W.; Davis, M.K.; Freed, D.H. Short-term mechanical circulatory support for recovery from acute right ventricular failure: Clinical outcomes. *J Heart Lung Transplant* **2014**, *33*, 794-799.
6. Coromilas, E.J.; Takeda, K.; Ando, M.; Cevasco, M.; Green, P.; Karmpaliotis, D.; Kirtane, A.; Topkara, V.K.; Yuzefpolskaya, M.; Takayama, H.; et al. Comparison of Percutaneous and Surgical Right Ventricular Assist Device Support After Durable Left Ventricular Assist Device Insertion. *J Card Fail* **2019**, *25*, 105-113.
7. Deschka, H.; Holthaus, A.J.; Sindermann, J.R.; Welp, H.; Schlarb, D.; Monsefi, N.; Martens, S.; Scherer, M. Can Perioperative Right Ventricular Support Prevent Postoperative Right Heart Failure in Patients with Biventricular Dysfunction Undergoing Left Ventricular Assist Device Implantation? *J Cardiothorac Vasc Anesth* **2016**, *30*, 619-626.
8. Jaidka, A.; De, S.; Nagpal, A.D.; Chu, M.W.A. Prophylactic Right Ventricular Assist Device for High-Risk Patients Undergoing Valve Corrective Surgery. *CJC Open* **2019**, *1*, 19-27.
9. Kapur, N.K.; Paruchuri, V.; Jagannathan, A.; Steinberg, D.; Chakrabarti, A.K.; Pinto, D.; Aghili, N.; Najjar, S.; Finley, J.; Orr, N.M.; et al. Mechanical circulatory support for right ventricular failure. *JACC Heart Fail* **2013**, *1*, 127-134.
10. Khani-Hanjani, A.; Loor, G.; Chamogeorgakis, T.; Shafii, A.; Mountis, M.; Hanna, M.; Soltesz, E.; Gonzalez-Stawinski, G.V. Case series using the rotaflow system as a temporary right ventricular assist device after heartmate II implantation. *ASAIO J* **2013**, *59*, 456-460.
11. Khors; i, M.; Schroder, J.; Daneshm; , M.; Bishawi, M.; Bouamra, O.; Winterton, P.; Choi, A.Y.; Patel, C.; et al. Outcomes After Extracorporeal Right Ventricular Assist Device Combined With Durable Left Ventricular Assist Device Support. *Ann Thorac Surg* **2019**, *107*, 1768-1774.
12. Kiernan, M.S.; Gr, W.; in, E.; Brinkley, M.; Kapur, N.K.; Pham, D.T.; Ruthazer, R.; Eduardo Rame, J.; Atluri, P.; Birati, E.Y.; et al. Early Right Ventricular Assist Device Use in Patients Undergoing Continuous-Flow Left Ventricular Assist Device Implantation: Incidence and Risk Factors from the Interagency Registry for Mechanically Assisted Circulatory Support. *Circ Heart Fail* **2017**, *10*.
13. Kremer, J.; Farag, M.; Brcic, A.; Zubarevich, A.; Schamroth, J.; Kreusser, M.M.; Karck, M.; Ruhparwar, A.; Schmack, B. Temporary right ventricular circulatory support following right ventricular infarction: results of a groin-free approach. *ESC Heart Fail* **2020**, *7*, 2853-2861.
14. Lazar, J.F.; Swartz, M.F.; Schiralli, M.P.; Schneider, M.; Pisula, B.; Hallinan, W.; Hicks Jr, G.L.; Massey, H.T. Survival after left ventricular assist device with and without temporary right ventricular support. *Ann Thorac Surg* **2013**, *96*, 2155-2159.

15. Leidenfrost, J.; Prasad, S.; Itoh, A.; Lawrance, C.P.; Bell, J.M.; Silvestry, S.C. Right ventricular assist device with membrane oxygenator support for right ventricular failure following implantable left ventricular assist device placement. *Eur J Cardio-thorac Surg* **2016**, *49*, 73-77.
16. Loforte, A.; Stepanenko, A.; Potapov, E.V.; Musumeci, F.; Dranishnikov, N.; Schweiger, M.; Montalto, A.; Pasic, M.; Weng, Y.; el, M.; et al. Temporary right ventricular mechanical support in high-risk left ventricular assist device recipients versus permanent biventricular or total artificial heart support. *Artif Organs* **2013**, *37*, 523-530.
17. Loforte, A.; Montalto, A.; Ranocchi, F.; Della Monica, P.L.; Casali, G.; Lappa, A.; Contento, C.; Musumeci, F. Levitronix CentriMag third-generation magnetically levitated continuous flow pump as bridge to solution. *ASAIO J* **2011**, *57*, 247-253.
18. Oliveros, E.; Collado, F.M.; Poulin, M.F.; Seder, C.W.; March, R.; Kavinsky, C.J. Percutaneous Right Ventricular Assist Device Using the TandemHeart ProtekDuo: Real-World Experience. *J Invasive Cardiol* **2021**, *33*, E407-E411.
19. Patil, N.P.; Mohite, P.N.; Sabashnikov, A.; Dhar, D.; Weymann, A.; Zeriouh, M.; Hards, R.; Hedger, M.; De Robertis, F.; Bahrami, T.; et al. Preoperative predictors and outcomes of right ventricular assist device implantation after continuous-flow left ventricular assist device implantation. *J Thorac Cardiovasc Surg* **2015**, *150*, 1651-1658.
20. Qureshi, A.M.; Turner, M.E.; O'Neill, W.; Denfield, S.W.; Aghili, N.; Badiye, A.; hi, R.; Tehrani, B.; Chang, G.; Oyama, J.K.; et al. Percutaneous Impella RP use for refractory right heart failure in adolescents and young adults—A multicenter U.S. experience. *Catheter Cardiovasc Interventions* **2020**, *96*, 376-381.
21. Ravich; ran, A.K.; Baran, D.A.; Stelling, K.; Cowger, J.A.; Salerno, C.T. Outcomes with the tandem protek duo dual-lumen percutaneous right ventricular assist device. *ASAIO J* **2018**, *64*, 570-572.
22. Saeed, D.; Maxhera, B.; Kamiya, H.; Lichtenberg, A.; Albert, A. Alternative right ventricular assist device implantation technique for patients with perioperative right ventricular failure. *J Thorac Cardiovasc Surg* **2015**, *149*, 927-932.
23. Saito, S.; Sakaguchi, T.; Miyagawa, S.; Nishi, H.; Yoshikawa, Y.; Fukushima, S.; Daimon, T.; Sawa, Y. Recovery of right heart function with temporary right ventricular assist using a centrifugal pump in patients with severe biventricular failure. *J Heart Lung Transplant* **2012**, *31*, 858-864.
24. Salna, M.; Garan, A.R.; Kirtane, A.J.; Karmpaliotis, D.; Green, P.; Takayama, H.; Sanchez, J.; Kurlansky, P.; Yuzefpolskaya, M.; Colombo, P.C.; et al. Novel percutaneous dual-lumen cannula-based right ventricular assist device provides effective support for refractory right ventricular failure after left ventricular assist device implantation. *Interact Cardiovasc Thorac Surg* **2020**, *30*, 499-506.
25. Schaefer, A.; Reichart, D.; Bernhardt, A.M.; Kubik, M.; Barten, M.J.; Wagner, F.M.; Reichenspurner, H.; Philipp, S.A.; Deuse, T. Outcomes of Minimally Invasive Temporary Right Ventricular Assist Device Support for Acute Right Ventricular Failure during Minimally Invasive Left Ventricular Assist Device Implantation. *ASAIO J* **2017**, *63*, 546-550.
26. Schmack, B.; Farag, M.; Kremer, J.; Grossekettler, L.; Brcic, A.; Raake, P.W.; Kreusser, M.M.; Goldwasser, R.; Popov, A.F.; Mansur, A.; et al. Results of concomitant groin-free percutaneous temporary RVAD support using a centrifugal pump with a double-lumen jugular venous cannula in LVAD patients. *J Thorac Dis* **2019**, *11*, S913-S920.
27. Schopka, S.; Haneya, A.; Rupprecht, L.; Camboni, D.; Schmid, C.; Hilker, M. Temporary right heart assist: A minimally invasive approach. *Artif Organs* **2012**, *36*, 700-704.

28. Shekiladze, N.; Condado, J.F.; esara, P.B.; Kim, J.H.; Devireddy, C.; McDaniel, M.; Babaliaros, V.; Samady, H.; Kumar, G.; Jaber, W.A. A single healthcare experience with Impella RP. *Catheter Cardiovasc Interventions* **2020**.
29. Takeda, K.; Naka, Y.; Yang, J.A.; Uriel, N.; Colombo, P.C.; Jorde, U.P.; Takayama, H. Outcome of unplanned right ventricular assist device support for severe right heart failure after implantable left ventricular assist device insertion. *J Heart Lung Transplant* **2014**, *33*, 141-148.
30. Vierecke, J.; Gahl, B.; De By, T.M.M.H.; Antretter, H.; Beyersdorf, F.; Caliskan, K.; Krachak, V.; Loforte, A.; Potapov, E.; Schoenrath, F.; et al. Results of primary biventricular support: An analysis of data from the EUROMACS registry. *Eur J Cardio-thorac Surg* **2019**, *56*, 1037-1045.
31. Yoshioka, D.; Takayama, H.; Garan, R.A.; Topkara, V.K.; Han, J.; Kurlansky, P.; Yuzefpolskaya, M.; Colombo, P.C.; Naka, Y.; Takeda, K. Contemporary outcome of unplanned right ventricular assist device for severe right heart failure after continuous-flow left ventricular assist device insertion. *Interact Cardiovasc Thorac Surg* **2017**, *24*, 828-834.
